# The role of hope, academic thriving, and adaptive coping in fostering peace of mind among university students: a mixed-methods study

**DOI:** 10.3389/fpsyg.2025.1510244

**Published:** 2025-08-18

**Authors:** Jing Shan, Teng Xu

**Affiliations:** ^1^College of Education and Modern Media, Shandong Institute of Petroleum and Chemical Technology, Dongying, Shandong, China; ^2^League Committee, Shandong University of Technology, Zibo, Shandong, China

**Keywords:** hope, academic thriving, adaptive coping, peace of mind, university students, emotional well-being, mixed-methods study, structural equation modeling

## Abstract

**Introduction:**

University students face significant academic stress, making psychological resources like hope, academic thriving, and adaptive coping essential for emotional well-being. Drawing on Snyder's Hope Theory and Schreiner's model of academic thriving, this study examines how these psychological constructs contribute to students' peace of mind.

**Methods:**

This mixed-methods study collected quantitative data from 562 undergraduate students in mainland China using validated measures of hope, academic thriving, adaptive coping, and peace of mind. Structural equation modeling (SEM) was used to analyze the quantitative data. Qualitative data were gathered through semi-structured interviews and reflective journals to provide additional context.

**Results:**

The quantitative analysis revealed that hope and academic thriving are both directly and indirectly associated with peace of mind, with adaptive coping playing a significant mediating role. Specifically, higher levels of hope and thriving predicted greater use of adaptive coping strategies, which, in turn, enhanced students' emotional tranquility and mental clarity. The qualitative data provided nuanced insights into how students' hope, thriving, and coping strategies shaped their experiences of peace of mind.

**Discussion:**

The findings suggest that promoting hope, fostering academic thriving, and teaching adaptive coping techniques can help students navigate academic challenges while maintaining emotional balance. These results have practical implications for educational interventions, highlighting the value of integrating psychological support into university settings to enhance both academic success and emotional well-being.

## 1 Introduction

University students increasingly face academic stress and psychological challenges (Gallagher et al., 2017; Pedrotti, 2018), necessitating that higher education institutions support emotional and psychological resilience alongside intellectual development (Brewer et al., 2019; Schreiner, 2010; Kristjánsson, 2016). Key psychological constructs—such as hope, academic thriving, and adaptive coping—are crucial for both academic achievement and emotional wellbeing (Carmona-Halty et al., 2021; Snyder et al., 2002; Skinner et al., 2013; Wong et al., 2024). Examining these constructs together offers insight into how students achieve psychological balance, specifically peace of mind, amidst higher education demands (Lee et al., 2013; Wu et al., 2020). Peace of mind, a core focus here, is comprehensively understood through its distinct definition rooted in Eastern philosophies, observable links to emotion regulation and physiological states, and cultivation via mindfulness practices (Halland et al., 2015; Lee et al., 2013; Xu et al., 2015).

Hope, defined by Snyder's Hope Theory 2002 as goal-oriented agency and pathways, fosters resilience and mitigates academic stress (Pedrotti, 2018; Rand et al., 2020), yet its direct link to peace of mind—characterized by emotional calmness and mental clarity—remains underexplored (Lee et al., 2013; Barcan, 2016; Yoon et al., 2015). Similarly, academic thriving, a multidimensional concept encompassing intellectual, emotional, and social success (Schreiner, 2010, 2017), equips students to manage stress and recover from setbacks (Kristjánsson, 2016; Rodríguez-Fernández et al., 2018), but its relationship with emotional outcomes like peace of mind is also not widely explored (Schreiner et al., 2020). Adaptive coping strategies (e.g., problem-solving, help-seeking, emotion regulation) are critical for managing academic stress and promoting emotional balance (Cao et al., 2021; Kao, 2024; Skinner et al., 2013; Vizoso et al., 2018), but their mediating role between hope, thriving, and peace of mind requires further examination.

This study addresses these gaps by exploring the connections between hope, academic thriving, adaptive coping, and peace of mind within the context of academic stress. It examines how these constructs interact to foster emotional wellbeing, specifically peace of mind, and introduces adaptive coping as a key mediator, offering a detailed understanding of the pathways through which psychological factors influence emotional outcomes. The study aims to examine the direct relationships between hope, academic thriving, and peace of mind, as well as the mediating role of adaptive coping in these relationships. Specifically, the study will test the following hypotheses: (1) Hope is directly related to peace of mind; (2) Academic thriving is directly related to peace of mind; (3) Adaptive coping mediates the relationship between hope and peace of mind; and (4) Adaptive coping mediates the relationship between academic thriving and peace of mind. By investigating these hypotheses, the study contributes empirical evidence on how psychological factors promote emotional wellbeing in academic contexts. The findings offer practical guidance for educators, counselors, and university administrators seeking to cultivate hope, academic thriving, and adaptive coping to enhance student wellbeing and resilience in response to academic challenges.

## 2 Review of the literature

### 2.1 The concept of hope in academic contexts

Hope is an essential psychological construct in educational contexts (Bozalek et al., 2014; Lucas et al., 2019). Snyder's (2002) Hope Theory defines it as a cognitive-motivational framework comprising goal-oriented agency (the perceived capacity to initiate and sustain action) and effective pathways (the perceived ability to devise strategies). These components work synergistically to maintain motivation and adapt when facing challenges (Fraser et al., 2022a; Snyder, 2000), proving valuable for academic resilience and success (Fraser et al., 2022b; Pedrotti, 2018).

As conceptualized by Snyder (2000), hope is a dynamic, future-oriented disposition and cognitive function, distinct from mere positive feeling or static personality traits, and not confined solely to its measurement. From a cognitive perspective, it engages executive functions like planning, problem-solving, and decision-making, as individuals envision future goals and devise strategies (Snyder, 2000; Snyder et al., 1991). While related to optimism, hope emphasizes active, goal-directed thinking and belief in one's capacity (Snyder, 2002; Snyder et al., 1991). Though it influences affective reactions by fostering positive emotions or mitigating anxiety, hope is fundamentally a cognitive-motivational construct rather than an emotional state (Gallagher et al., 2017). It provides unique motivational impetus through its 'will and ways' components, exhibiting both trait-like stability and state-like variability that allows it to be shaped by situational factors (Snyder et al., 1991).

Hope acts as a psychological resource, helping students manage academic stress while pursuing long-term goals (Gallagher et al., 2017; Griggs, 2017). Students with high hope levels are more likely to perceive academic stressors as challenges rather than threats, fostering a proactive approach to stress management (Rand et al., 2020; Yoon et al., 2015). This reframing enhances engagement, enabling sustained effort during high-stress periods, which supports both academic persistence and wellbeing (Marques et al., 2017; Pedrotti, 2018). Furthermore, hope's pathways component equips students with the flexibility to adjust strategies when encountering obstacles, enhancing their resilience in demanding academic environments (Snyder et al., 2002).

Research consistently shows that hope predicts academic success. Studies link hope to positive outcomes like higher grade point averages, motivation, and engagement (Marques et al., 2017; Yotsidi et al., 2018). Hope encourages students to set ambitious goals and persevere, even when facing difficulties (Levi et al., 2014; Marques et al., 2017). High-hope students also tend to engage in proactive behaviors, such as seeking help from teachers or peers, which contributes to both academic success and emotional wellbeing (Bryce et al., 2020; Dixson and Gentzis, 2023).

It is important to clearly distinguish hope from coping skills. Hope is not a coping strategy itself, but rather a precursor and energizer for adaptive coping behaviors (Folkman, 2012; Henry, 2004). Hope provides the 'why' (motivation for goals) and the “what” (belief in strategies), making students more likely to actively utilize constructive approaches like problem-solving and seeking social support to handle academic stress (Fraser et al., 2022a; Pedrotti, 2018; Snyder, 2002). For example, a hopeful student, believing in their ability to find solutions, is more inclined to use active coping strategies than a less hopeful student who might resort to avoidance (Rand et al., 2020). This fosters their ability to view academic challenges as temporary and manageable setbacks, thereby indirectly supporting overall psychological wellbeing and emotional regulation (Gallagher et al., 2017; Rand et al., 2020).

Compared to other psychological constructs, hope is distinct. It is a motivational state, unlike general wellbeing which is an outcome (Pedrotti, 2018). Unlike compassion, which is outwardly focused on others' suffering, hope is self-directed toward personal goal achievement (Howell and Larsen, 2015). While locus of control shares similarities with hope's agency component, hope uniquely incorporates the pathways component, emphasizing strategic thinking (Bernardo, 2010; Snyder, 2000). Self-transcendence, involving a focus beyond the self, differs from hope's emphasis on personal goals (Snyder, 2002; Snyder et al., 1991).

In the context of mainland China, cultural values significantly shape the expression and perceived importance of hope among university students (Zhang et al., 2015). Chinese society exhibits a paradox where confidence in the nation's future often coexists with personal uncertainty, reflecting the complex interplay between collective and individual aspirations (Linyan and Boqing, 2023). Collectivist cultures may emphasize hope for collective wellbeing and fulfilling family or societal expectations (Bernardo et al., 2018). The intense academic pressure and rapid socio-economic changes in China further amplify the need for robust psychological resources like hope to navigate uncertainty and maintain focus on future goals (Liang et al., 2020; Zhao et al., 2015). Therefore, understanding hope within this specific educational and cultural backdrop is crucial.

Despite hope's well-established positive effects on academic outcomes and resilience, research on its role in fostering peace of mind—a state of emotional tranquility and mental clarity—remains limited. While hope buffers against academic stress (Rand et al., 2020; Wong and Cheung, 2024), few studies examine its contribution to emotional balance, particularly in relation to peace of mind (Lee et al., 2013). Given that peace of mind is a valuable outcome in academic settings, further research is needed to explore how hope supports this aspect of wellbeing.

In conclusion, hope is a multidimensional cognitive-motivational construct with important implications for academic performance and psychological health. Its influence extends beyond motivating academic success to promoting adaptive coping strategies that support mental wellbeing. The fundamental human capacity for goal-directed thought and action suggests a universal basis for hope, transcending cultural boundaries (Snyder, 2000). While specific goals and preferred pathways may vary, the underlying mechanism of hopeful thinking—envisioning and pursuing desired futures—remains a common psychological process (Fraser et al., 2022a; Cheavens et al., 2006). Considering its significant role in academic resilience, motivation, and emotional balance, hope is a key psychological resource for students navigating higher education.

### 2.2 Academic thriving: a holistic construct

Academic thriving is a multidimensional construct extending beyond traditional academic performance to encompass intellectual, emotional, and social dimensions contributing to overall student wellbeing (Schreiner, 2010, 2017). Defined by Schreiner (2010) through five key components—engaged learning, academic determination, social connectedness, positive perspective, and diverse citizenship—this framework integrates cognitive and non-cognitive factors, suggesting thriving students develop psychological resilience and social engagement (Cuevas et al., 2017; Schreiner et al., 2020). Distinct from general wellbeing, which spans multiple life domains, academic thriving specifically captures optimal functioning and flourishing within the academic environment (Cuevas, 2015; Gereluk, 2018). It integrates performance (e.g., grades) with psychological and social indicators (e.g., motivation, belonging) directly tied to educational success (Schreiner, 2017; Kristjánsson, 2016; Schreiner, 2010; Cuevas et al., 2017). This context-specific integration highlights thriving as a dynamic process emphasizing active student development and adaptation within their educational journey (Green and Rizwan, 2024).

Research consistently shows academic thriving predicts both achievement and personal wellbeing. Thriving students engage deeply in learning, fostering intrinsic motivation and persistence (Cuevas et al., 2017; Green and Rizwan, 2024; Kristjánsson, 2016). This deep engagement links to long-term academic success beyond grades (Van Zyl and Rothmann, 2012). Thriving also enhances resilience, enabling effective stress management and recovery from setbacks (Kotzé and Kleynhans, 2013; Morgan Consoli et al., 2015). Social connectedness, a key component, offers emotional support and a sense of belonging (Rodríguez-Fernández et al., 2018; Sayler, 2009). This holistic approach, rooted in Aristotelian philosophy, positions thriving as a dynamic process of growth and adaptation, where students fully engage in their academic and social environments, leading to higher life satisfaction and psychological wellbeing (Jaleel et al., 2024; Kristjánsson, 2016; Van Zyl and Rothmann, 2012; Cuevas et al., 2017). The Academic Thriving Questionnaire (Schreiner et al., 2009) operationalizes this distinct construct by measuring its integrated components within the academic context, ensuring its clarity and measurability as a unique construct (Schreiner, 2010; Schreiner et al., 2009).

Although strongly linked to academic success and resilience, research on how thriving relates to adaptive coping strategies remains limited. While thriving students manage academic stress effectively, the specific mechanisms through which they employ coping strategies—like problem-solving or seeking support—are underexplored (Morgan Consoli et al., 2015; Schreiner, 2010; Rodríguez-Fernández et al., 2018). Understanding these mechanisms is crucial for developing interventions. In summary, academic thriving integrates intellectual, emotional, and social aspects of student success, with Schreiner's (2010) model emphasizing non-cognitive factors. As a specific form of wellbeing tied to educational experience, thriving's association with success and resilience calls for future research to explore adaptive coping's role in supporting emotional wellbeing and peace of mind, thereby comprehensively understanding how thriving fosters both academic success and long-term mental health.

### 2.3 Adaptive coping strategies and academic stress

Adaptive coping refers to strategies individuals employ to manage stress and navigate challenges effectively (Thompson et al., 2010; Zeidner and Saklofske, 1996). Crucially, a coping mechanism's adaptive nature is not intrinsic; it depends on the person, context, and outcomes (Ntoumanis et al., 2009; Zeidner and Saklofske, 1996). In academic settings, strategies are generally considered adaptive if they lead to constructive outcomes like problem resolution, enhanced wellbeing, or sustained engagement, rather than emotional withdrawal (Gonçalves et al., 2019; Skinner and Saxton, 2019).

Skinner et al. (2013) identify key strategies often adaptive in academic contexts: problem-solving, help-seeking, and emotional regulation. These foster resilience, preventing stress from undermining performance and wellbeing (Skinner and Saxton, 2019; Vizoso et al., 2018). Students effectively employing these strategies tend to show increased engagement, persistence, and emotional stability under pressure (Skinner et al., 2016; Wu et al., 2020). However, effectiveness varies individually; problem-solving benefits analytical students, help-seeking suits those with strong social networks (Malkoç and Yalçin, 2015), and emotional regulation aids those facing high emotional stress (Zeidner and Saklofske, 1996).

Research consistently demonstrates that students using adaptive coping achieve greater academic success. Problem-solving and help-seeking enhance motivational resilience, fostering commitment despite challenges (Skinner et al., 2016). These constructive approaches mitigate stress's negative effects on academic performance and mental health (Enns et al., 2018). For instance, Konaszewski et al. (2021) found self-efficacy and sense of coherence predicted better academic outcomes and lower perceived stress. Cognitive restructuring, another adaptive strategy, improves academic engagement by reframing negative thoughts into positive perspectives (Vizoso et al., 2018). This approach enhances resilience, contributes to improved academic outcomes (Chen, 2016), and helps prevent burnout (Malkoç and Yalçin, 2015).

Adaptive coping also critically supports psychological wellbeing. Effective mechanisms correlate with lower anxiety, depression, and distress, alongside higher life satisfaction and resilience (Gloria and Steinhardt, 2016; Rahat and Ilhan, 2016). Wu et al. (2020) found positive coping styles linked to psychological resilience among Chinese university students, suggesting proactive stress management reduces negative emotional consequences. These findings align with broader research: adaptive coping enhances mental health and academic persistence (Eden et al., 2020). Emotional regulation is a core adaptive aspect, helping students manage emotional responses to stressors. Mindfulness training, for example, improves problem-focused coping and reduces academic stress (Halland et al., 2015). Effective emotional regulation reduces reactivity and catastrophizing, leading to productive coping behaviors and overall wellbeing (Skinner et al., 2013).

In the Chinese academic context, cultural and societal factors influence coping strategies (Hsu et al., 2008). Traditional emphasis on academic success, filial piety, and collective harmony (Cao et al., 2021) may prioritize strategies like persistence, self-reliance, and seeking support from close groups. Pressure to conform and maintain 'face' might encourage private emotional regulation or indirect help-seeking (Chen, 2016; Ma et al., 2022). Understanding these contextual nuances is crucial for interpreting stress management in this setting.

Despite adaptive coping's benefits, its mediating role between hope, academic thriving, and peace of mind remains underexplored. Hope theory suggests hopeful individuals are more likely to engage in goal-directed problem-solving (Snyder et al., 2002), implying adaptive coping mediates hope's influence on academic success and emotional wellbeing. Similarly, academic thriving, linked to positive outcomes (Schreiner, 2010), likely uses adaptive coping to sustain engagement and persistence under stress (Vizoso et al., 2018). Understanding this mediation offers deeper insights into how students achieve emotional balance and resilience in academic environments.

### 2.4 Peace of mind: emotional and psychological wellbeing

Peace of mind (PoM), defined by (Lee et al. 2013) as emotional tranquility and mental clarity, is increasingly recognized as a key component of psychological wellbeing, especially in academic contexts (Datu, 2017; Liao et al., 2023). This state is characterized not by the absence of external challenges, but by an internal capacity for a calm psychological state and clear cognitive function despite environmental demands (Rachmad, 2001). It is a stable, low-arousal emotional state, distinctly focusing on inner harmony (Lee et al., 2013).

To clarify its specificity, PoM is crucial to distinguish from other psychological constructs. While contributing to overall wellbeing, PoM is not synonymous with general wellbeing (a broader concept encompassing various life domains and positive emotions, some high-arousal), nor with fleeting happiness (Sophie et al., 2022). In contrast, PoM focuses on achieving a sustained state of inner emotional balance and mental lucidity, which supports resilience in academic challenges by enabling students to maintain focus and clarity even during high-pressure situations (Datu, 2017; Xu et al., 2015). Cognitively, PoM involves processes supporting emotional regulation and attentional control (Xu et al., 2015); affectively, it is marked by low-arousal positive emotions like calmness and serenity, distinguishing it from high-arousal states (Lee et al., 2013). As an experiential state, PoM fluctuates rather than representing a fixed personality trait.

Furthermore, PoM is distinct from personality traits or other dispositions like locus of control, or prosocial orientations such as compassion and self-transcendence. While an internal locus of control (the belief that one controls one's own destiny) may facilitate a sense of calm by promoting agency, PoM is the resulting emotional experience rather than the belief system itself (Bernardo, 2010). Compassion is an outward-directed emotion of concern for others' suffering, and self-transcendence involves moving beyond self-interest to connect with something larger. While these constructs can foster a sense of inner peace, PoM specifically describes an individual's internal state of calm and cognitive stability, regardless of its source (Miller and Verhaeghen, 2022; Steger, 2013).

In contrast to flow, which involves deep engagement in an activity, or mindfulness, which emphasizes present-moment awareness, PoM specifically denotes a sustained sense of inner calm and mental clarity, often irrespective of external engagement (Csikszentmihalyi, 1990; Lee et al., 2013; Wang and Tang, 2025). It is not confined to the structure of its measurement instruments but represents a deeply experienced psychological state (Rachmad, 2001). While adaptive coping strategies—such as mindfulness or problem-solving—can facilitate PoM, the state of PoM itself is the outcome of effective emotional and cognitive regulation, not the coping behavior *per se* (Skinner et al., 2013; Xu et al., 2015).

The theoretical grounding for peace of mind (PoM) is enriched by insights from diverse disciplines and philosophical traditions (Misra, 2023). The emphasis of PoM on internal harmony and emotional balance is deeply rooted in Eastern philosophies (Lee et al., 2013). This contrasts sharply with Western cultural emphasis on high-arousal emotions and external achievement. While Eastern thought prioritizes inner harmony, acceptance of impermanence, and emotional regulation for wellbeing, the human desire for inner calm is universal, even if its expression and cultural prioritization vary (Lee et al., 2013; Steger, 2013; Yu et al., 2020). Thus, the ability to maintain composure and mental clarity amidst stressors serves as a fundamental, transcultural psychological resource.

From an affective neuroscience perspective, peace of mind is hypothesized to involve specific neural correlates (Misra, 2023). This state correlates with enhanced functional connectivity within prefrontal cortical networks that support cognitive control and emotional appraisal (Gloria and Steinhardt, 2016; Ochsner and Gross, 2005). Concurrently, deep calm often involves down-regulation of amygdala activity, the brain region central to fear processing (Deshmukh, 2022). Physiologically, a shift toward parasympathetic nervous system dominance contributes to bodily calm. Furthermore, reduced activity in the default mode network (DMN), typically active during rumination, may underpin mental clarity and reduced distraction (Raichle et al., 2001). While direct neuroimaging of “peace of mind” is an emerging area, related research on emotion regulation and stress reduction provides strong theoretical support for these mechanisms.

The cultivation of peace of mind is also strongly linked to mindfulness literature and its practices (Xu et al., 2015). Mindfulness, defined as paying attention on purpose, in the present moment, and non-judgmentally, directly fosters PoM components (Kabat-Zinn, 1990; Savel and Munro, 2017). Practices training present-moment awareness and non-reactive observation lead to reduced emotional reactivity, increased mental clarity, and inner calm (Xie, 2023; Xu et al., 2015). Research on mindfulness training shows significant improvements in problem-focused coping and reduced academic stress (Halland et al., 2015), directly supporting emotional tranquility pathways inherent in PoM (Savel and Munro, 2017). By cultivating mindful awareness, individuals better regulate emotions, fostering the internal balance defining peace of mind (Shapiro et al., 2006).

In academic settings, PoM has been less frequently studied than constructs like happiness or life satisfaction (Sophie et al., 2022), yet it is highly relevant for students facing fluctuating stress and anxiety. Achieving PoM enables students to maintain focus and clarity even during high-pressure situations (Datu, 2017). Emerging evidence highlights its importance for student wellbeing: PoM is positively associated with academic motivation and achievement (Datu, 2017), and linked to emotional regulation (Xu et al., 2015). Students with higher self-acceptance and mindfulness tend to report greater PoM, which aids in more effective academic stress coping (Xu et al., 2015).

PoM provides a stable emotional foundation, fostering resilience in demanding academic environments. In high-stakes settings, students with PoM are better equipped to handle pressures without emotional burnout (Liang et al., 2020). Research indicates PoM buffers against stress by promoting adaptive coping strategies, such as problem-solving and help-seeking (Liao et al., 2023), contributing to both short-term academic performance and long-term psychological health. Students experiencing PoM are less prone to mental health issues like anxiety and depression (Xu et al., 2015). Beyond immediate stress management, PoM enhances emotional stability and resilience, leading to improved academic engagement and success (Datu et al., 2018; Yu et al., 2020). For example, resilience has been shown to mediate the relationship between physical activity and PoM (Liao et al., 2023), and PoM mediates the link between gratitude and reduced depression, underscoring its protective role (Liang et al., 2020).

Despite these findings, a gap persists regarding PoM's role in academic settings, particularly its connection to hope and academic thriving. Few studies have explored how these constructs contribute to emotional balance in high-pressure environments (Datu et al., 2018; Yu et al., 2020). Cross-cultural studies are also needed to understand how PoM is valued, given that East Asian individuals often score higher on the PoMS than Western counterparts (Lee et al., 2013). Understanding these nuances is crucial for developing effective interventions across diverse student populations. Overall, PoM, characterized by emotional tranquility and mental clarity, is vital for understanding psychological wellbeing in academic contexts. This distinct internal state supports sustained cognitive function and emotional regulation. Although research remains limited, achieving PoM helps students manage stress, build resilience, and contributes to long-term mental health. Further exploration of how hope and thriving influence PoM, alongside cross-cultural comparisons, is essential for future research. Promoting PoM holds significant potential for enhancing both academic performance and emotional wellbeing.

### 2.5 The objectives of the study

This study investigates the relationships and associations between hope, academic thriving, adaptive coping, and peace of mind in the academic context. Drawing from established theories and empirical studies, it examines how hope and academic thriving relate to students' emotional wellbeing, specifically their sense of peace of mind. Peace of mind, defined as a stable state of emotional tranquility and mental clarity (Lee et al., 2013), and distinct from other forms of wellbeing, is essential for helping students manage academic stress while maintaining emotional balance. The study explores both the direct effects of hope and thriving and the mediating role of adaptive coping in these relationships.

The quantitative part of the research addresses four main hypotheses. The first hypothesis (H1) posits that hope is directly related to peace of mind. Building on Snyder's Hope Theory 2002, which highlights the importance of agency and pathways in goal pursuit, we hypothesize that students' capacity to define goals and devise strategies fosters a positive outlook and emotional stability that directly contributes to their sense of inner calm. This cognitive-motivational framework, which directs active coping efforts, helps students maintain a positive outlook and emotional stability during academic stress (Gallagher et al., 2017). Hopeful students tend to see academic challenges as manageable, which fosters resilience and contributes to peace of mind (Pedrotti, 2018).

The second hypothesis (H2) proposes that academic thriving is also directly linked to peace of mind. Drawing from Schreiner's (2010) model of thriving, which integrates intellectual, emotional, and social dimensions of student success, we hypothesize that students who are deeply engaged in learning and socially connected will experience enhanced psychological resilience and a greater sense of emotional balance. It represents a specific form of wellbeing tied to academic pursuits, where students demonstrate optimal functioning and growth within their educational context. Thriving students develop intellectual, social, and emotional resilience, helping them manage academic pressures and enhance their emotional wellbeing (Rodríguez-Fernández et al., 2018). This study explores how thriving contributes to peace of mind through academic and social success.

The third hypothesis (H3) explores whether adaptive coping mediates the relationship between hope and peace of mind. Consistent with problem-focused coping theories (Skinner et al., 2013) and aligning with Snyder's Hope Theory, which suggests that hopeful individuals are more proactive in overcoming obstacles, we hypothesize that adaptive coping strategies serve as the mechanism through which hope translates into peace of mind. Adaptive coping strategies, such as problem-solving and emotional regulation, are commonly used by hopeful individuals to manage stress effectively (Skinner et al., 2013). These strategies help maintain emotional balance, suggesting that adaptive coping mediates the link between hope and peace of mind (Wu et al., 2020).

The fourth hypothesis (H4) examines whether adaptive coping mediates the relationship between academic thriving and peace of mind. Based on Schreiner's (2010) holistic view of thriving, which implies active engagement and resilience, and supported by models of effective stress management (Vizoso et al., 2018), we hypothesize that thriving students' greater use of adaptive coping strategies explains their enhanced peace of mind. Thriving students are more likely to use adaptive coping strategies, which help them manage stress and maintain emotional stability (Schreiner, 2010). Understanding this mediation is key to recognizing how thriving supports both academic success and long-term emotional wellbeing (Vizoso et al., 2018).

In addition to the quantitative analysis, the study incorporates a qualitative component that explores students' lived experiences with academic stress and how hope, academic thriving, and adaptive coping contribute to their peace of mind. Using semi-structured interviews and reflective journals, the study delves into personal narratives to reveal the psychological resources students rely on when facing academic challenges. This qualitative approach provides deeper insights into the complex relationships between these constructs, highlighting individual strategies for emotional resilience and wellbeing.

## 3 Methodology

### 3.1 Sample

A total of 562 full-time undergraduate students from four comprehensive universities across mainland China were recruited for this study. These universities were purposefully selected to represent a diverse range of institutional types, including urban and rural campuses, and to ensure variation in student demographics, academic disciplines, and regional contexts. Participants came from a variety of fields such as engineering, social sciences, and humanities, reflecting the wide range of academic environments and pressures experienced by students in these disciplines. This diverse recruitment strategy aimed to capture the broad academic experience across the general university student population in China. The average age of participants was 20.2 years (SD = 1.5), with ages ranging from 18 to 24. Gender distribution was relatively balanced, with 287 females (51%) and 275 males (49%), consistent with the gender composition typically found in Chinese universities.

Inclusion criteria for participants were clearly defined: full-time undergraduate students enrolled in their second or third year of study at one of the participating universities. This specific criterion was employed because students in these years are often deeply immersed in their academic programs and likely to have developed coping mechanisms for the ongoing demands of university life. First-year students were excluded to avoid variability caused by the adjustment period to university life, and fourth-year students were excluded due to their heightened focus on graduation requirements and post-university plans, which could have influenced their levels of academic stress and thriving. No prior experience with stress management techniques or positive psychology interventions was required for inclusion, ensuring a naturalistic sample.

Recruitment was carried out using convenience sampling. Initial contact was made through university faculty members in relevant departments who shared information about the study with their students, and through university student organizations which disseminated announcements. Announcements were also made via email, class forums, and social media platforms specific to the universities. Additionally, posters were placed in common student areas, such as libraries and dormitories, to encourage broad participation. All students who expressed interest in participating were directed to an online portal where they could access detailed information about the study, including its objectives, procedures, and ethical considerations. Participation was entirely voluntary, and a small incentive—a certificate for extracurricular activity credit—was offered to encourage participation without introducing bias.

The sample size of 562 for the quantitative component was determined to ensure sufficient statistical power for Structural Equation Modeling (SEM) analyses, particularly for detecting hypothesized mediation effects. Based on recommendations for complex models, a sample size of over 200 is generally considered adequate for stable parameter estimates and reliable fit indices (Kline, 2016; Hu and Bentler, 1999). Our final sample size well exceeded this recommendation, providing robust power for our analyses.

Informed consent was obtained electronically, with participants required to review a detailed consent form and affirm their understanding before beginning the survey. The consent form outlined their right to withdraw from the study at any point without any repercussions to their academic standing. Full ethical approval for this study was granted by the Institutional Review Board (IRB) of Shandong University of Technology (Reference Number: SUT-IRB-2023-A072-PSY). The research strictly adhered to ethical guidelines, ensuring participant confidentiality. All personal identifiers were anonymized in the dataset, and only the research team had access to the raw data.

Special attention was given to minimizing any potential psychological discomfort that participants might experience, particularly during the qualitative interviews and reflective journal entries. Since some participants may have reflected on stressful academic experiences, they were reminded that they could skip any questions that made them uncomfortable or stop their participation at any time. Additionally, contact information for university counseling services was provided to all participants, offering them access to mental health support if needed.

### 3.2 Data collection tools

To assess the key constructs of hope, adaptive coping, academic thriving, and peace of mind, we employed a combination of quantitative scales and qualitative data collection methods, including semi-structured interviews and reflective journals. Each scale was translated and back-translated to ensure linguistic accuracy and cultural appropriateness for Chinese university students. A pilot study with 50 participants was conducted to assess content and face validity, followed by confirmatory factor analyses (CFAs) for each scale to confirm construct validity. All scales demonstrated strong internal consistency, with Cronbach's alpha values ranging from 0.84 to 0.88.

#### 3.2.1 Hope

The Dispositional Hope Scale (DHS; Snyder et al., 1991) was utilized to evaluate participants' levels of hope. This instrument comprises 12 self-report items, designed to capture two key components of hope: agency (the perceived ability to initiate and sustain actions toward goals) and pathways (the perceived capacity to generate routes to achieve those goals). Participants rated their agreement with statements such as “I can think of many ways to get out of a jam” on an 8-point Likert scale, ranging from 1 (definitely false) to 8 (definitely true). Higher scores indicate greater dispositional hope. CFA results indicated good construct validity for the hope scale: χ^2^/df = 2.14, CFI = 0.95, TLI = 0.94, RMSEA = 0.048 (CI:0.038 −0.058), SRMR = 0.032, with Cronbach's alpha = 0.88.

#### 3.2.2 Adaptive Coping Styles

The Academic Coping Styles Questionnaire, developed by Skinner et al. (2013), was employed to assess participants' use of adaptive coping strategies when facing academic challenges. The questionnaire comprises 30 items, covering five distinct adaptive coping styles: strategy-building, commitment, self-motivation, seeking comfort, and seeking help. Responses were recorded using a 5-point Likert scale, where participants indicated their level of agreement from 1 (strongly disagree) to 5 (strongly agree). CFA results for the adaptive coping scale indicated good construct validity: χ^2^/df = 2.25, CFI = 0.93, TLI = 0.92, RMSEA = 0.049 (CI:0.041 −0.057), SRMR = 0.035, with Cronbach's alpha = 0.84.

#### 3.2.3 Academic thriving

Academic thriving was measured using the 18-item Academic Thriving Questionnaire, developed by Schreiner et al. (2009). This instrument captures five critical dimensions of academic success: engaged learning, academic determination, positive perspective, social connectedness, and diverse citizenship. Participants rated items on a 6-point Likert scale, from 1 (completely disagree) to 6 (completely agree). Higher scores suggest a greater degree of thriving in the academic environment. The questionnaire has shown excellent reliability in previous research, with a reported Cronbach's alpha of 0.98. CFA results confirmed construct validity for the academic thriving scale: χ^2^/df = 2.18, CFI = 0.94, TLI = 0.93, RMSEA = 0.046 (CI:0.037–0.055), SRMR = 0.033, with Cronbach's alpha = 0.85.

#### 3.2.4 Peace of mind

Participants' peace of mind was assessed using the Peace of Mind Scale (PoMS) developed by Lee et al. (2013). This scale consists of seven items, designed to evaluate the extent to which individuals experience peace and mental harmony in their daily lives. Participants responded to statements such as “I have peace and harmony in my mind” on a 5-point Likert scale, ranging from 1 (not at all) to 5 (all of the time). Higher scores on the PoMS reflect greater levels of peace of mind, with higher values indicating a state of calm and balance amidst academic stressors. CFA results for the peace of mind scale also showed good construct validity: χ^2^/df = 2.09, CFI = 0.96, TLI = 0.95, RMSEA = 0.044 (CI:0.035–0.053), SRMR = 0.030, with Cronbach's alpha = 0.87.

#### 3.2.5 Semi-structured interviews

To complement the quantitative findings, semi-structured interviews were conducted to explore participants' lived experiences with academic stress, coping strategies, hope, and academic thriving. An interview guide was developed, comprising 10 open-ended questions aimed at eliciting detailed responses. Example questions included, “Can you describe a recent academic challenge you faced, and how you approached it?” and “How does your sense of hope affect the way you deal with academic stress?” Follow-up questions were used to encourage participants to elaborate on specific instances or emotions they experienced during high-stress periods, such as exams or project deadlines.

Interviews lasted between 30 and 45 min, depending on the depth of participants' responses and their comfort level in discussing personal experiences. For instance, one participant, a third-year engineering student, recounted how a particularly difficult semester led to feelings of burnout, but hope in future academic success helped them persist through the challenges. Another participant, studying social sciences, detailed how they sought academic and emotional support from peers and professors when overwhelmed with coursework, demonstrating the interplay between hope and social connectedness.

Interviews were conducted by trained research assistants, either in person in a quiet, private on-campus office or remotely via Zoom for participants who preferred online participation due to scheduling or travel constraints. All interviews were audio-recorded, transcribed verbatim, and analyzed thematically.

#### 3.2.6 Reflective journals

In addition to interviews, participants were invited to submit reflective journals, offering a written narrative of their personal experiences with academic stress and coping strategies over the course of the semester. Prompts were designed to encourage deep reflection, with questions such as, “Describe a time when academic pressures felt overwhelming and how you managed them,” and “In what ways did hope or optimism help you navigate academic challenges?” Participants were asked to write about both successful and less effective coping strategies and to reflect on how their academic thriving influenced their sense of wellbeing.

Participants were given a week to complete their journal entries, allowing them to thoughtfully consider their experiences and provide thorough reflections. One student, for example, shared how they had developed a personal routine of mindfulness and exercise to manage anxiety during exams, while another discussed their use of goal-setting techniques rooted in their sense of hope to break down larger academic tasks into manageable steps. These reflective accounts offered a rich, nuanced view of individual coping mechanisms, adding depth to the quantitative analysis.

The journals were submitted electronically through a secure platform, and thematic analysis, using both open and axial coding, was applied to categorize common patterns and themes. These qualitative themes, such as emotional resilience, the role of social support, and the impact of hope on stress management, were then integrated with the quantitative data during the final interpretation and discussion, allowing for a more holistic understanding of the interactions between hope, thriving, adaptive coping, and peace of mind.

### 3.3 Research procedures

Data collection took place over an eight-week period during the spring semester, ensuring participants were actively experiencing academic stressors. Recruitment efforts were conducted across four universities to gather a diverse sample. The research team collaborated with academic departments and student organizations. Faculty members announced the study during lectures, and student representatives shared recruitment flyers on popular social media platforms like WeChat and QQ. Physical flyers were posted in high-traffic areas such as dining halls and dormitories, and email invitations were sent through university mailing lists. Interested participants accessed the study's webpage on a secure server, which provided details on the study's objectives, procedures, and ethical safeguards. Informed consent was obtained electronically, and participants were assured of confidentiality and the right to withdraw at any time.

Data collection occurred in two phases. In the first phase, participants completed an online survey consisting of validated self-report questionnaires. The survey, accessible on both mobile devices and computers, took 25–35 min to complete. Participants were encouraged to answer honestly, as there were no right or wrong responses. Upon completing the survey, participants were invited to participate in the qualitative phase, which included either a semi-structured interview or a reflective journal submission, based on their preference. Interviews, conducted by trained research assistants either in-person or via Zoom, lasted 30–45 min. They focused on participants' experiences with academic stress, coping strategies, and the role of hope and thriving in maintaining emotional wellbeing.

The participants who chose to submit reflective journals received prompts encouraging self-reflection on academic challenges, coping strategies, and how hope and thriving influenced their peace of mind. Journals were submitted electronically within a week of survey completion. Weekly email reminders were sent to participants to ensure survey and journal completion. A total of 562 students completed the quantitative survey, with 30 participating in the qualitative phase through interviews or journal submissions. The qualitative sample size (N=30) was deemed appropriate for thematic analysis to achieve saturation of themes and provide rich, in-depth insights into participants' lived experiences, consistent with recommendations for qualitative research (Creswell and Poth, 2018; Morse, 2015).

### 3.4 Data analysis

Quantitative data were analyzed using SPSS (Ver. 26.0) and AMOS (Ver. 24.0) to test the hypothesized mediation model. Descriptive statistics were computed for all key variables to assess central tendencies and variability. Cronbach's alpha values for all scales exceeded 0.70, indicating acceptable internal consistency (Field, 2013).

Structural Equation Modeling (SEM) was employed to assess the hypothesized relationships between the variables. Given the cross-sectional nature of our data, it is important to note that while SEM allows for the testing of theoretical pathways and mediation effects, these findings indicate associations and do not establish direct causality. The model posited that hope and academic thriving would predict peace of mind, with adaptive coping functioning as a mediator. Several fit indices were used to evaluate the adequacy of the model, including the Comparative Fit Index (CFI), Tucker-Lewis Index (TLI), Root Mean Square Error of Approximation (RMSEA), and Standardized Root Mean Square Residual (SRMR). Acceptable thresholds were set based on established guidelines (CFI and TLI ≥ 0.90, RMSEA and SRMR ≤ 0.08) (Hu and Bentler, 1999; Kline, 2016). The mediation analysis was further refined using bootstrapping methods with 5,000 resamples to determine the significance of indirect effects (Preacher and Hayes, 2008). The results indicated that both hope and academic thriving had significant direct effects on peace of mind, while adaptive coping partially mediated these relationships, providing support for the theoretical model.

Thematic analysis of the qualitative data was performed using NVivo, following Braun and Clarke's (2006) six-step process: familiarization, coding, theme identification, review, definition, and report writing. Open coding identified patterns related to coping strategies, hope, and peace of mind, which were then categorized into broader themes through axial coding. Selective coding refined central themes consistent with the quantitative findings, particularly regarding the mediating role of adaptive coping. To ensure rigor, member checking was conducted, allowing participants to review the initial themes for accuracy (Morse, 2015). Peer debriefing sessions with two external researchers further ensured the robustness and impartiality of the analysis (Creswell and Poth, 2018). The integration of qualitative and quantitative strands occurred primarily at the interpretation and discussion phase (e.g., in Section 4.2 and Section 5), where qualitative narratives provided nuanced insights that enriched and contextualized the statistical findings, offering a more holistic understanding of the phenomena.

## 4 Results

### 4.1 Quantitative results

#### 4.1.1 Descriptive statistics

A total of 562 participants completed the quantitative survey, with descriptive statistics for hope, academic thriving, adaptive coping, and peace of mind presented in Table 1. To address missing data, Full Information Maximum Likelihood (FIML) was employed due to its effectiveness for data assumed to be Missing at Random (MAR). FIML facilitated parameter estimation by leveraging all available data, thereby retaining the full sample and statistical power. Missing data were minimal (< 5% per item), and sensitivity analyses confirmed result consistency across different imputation methods.

**Table 1:** Descriptive statistics (*N* = 562).

**Variable**	**Mean**	**SD**	**Min**	**Max**	**Range**
Hope (DHS)	5.76	1.12	1.25	8.00	6.75
Academic Thriving	4.85	0.93	2.10	6.00	3.90
Adaptive Coping	4.25	0.85	2.10	5.00	2.90
Peace of Mind	3.88	0.92	1.75	5.00	3.25

Mean scores indicated moderate to high levels for all constructs. Hope averaged 5.76 (*SD* = 1.12), reflecting moderate-to-high goal-setting confidence consistent with its agency and pathways components (Snyder et al., 1991). Academic thriving had a mean of 4.85 (*SD* = 0.93, range: 2.10–6.00), suggesting positive academic experiences with some variability. Adaptive coping averaged 4.25 (*SD* = 0.85, range: 2.10–5.00), indicating moderate use of strategies like problem-solving and help-seeking. Peace of mind had a mean of 3.88 (*SD* = 0.92, range: 1.75–5.00), reflecting moderate emotional tranquility despite reported academic pressures by some.

#### 4.1.2 Correlation analysis

A Pearson correlation matrix, presented in Table 2, examined relationships among hope, academic thriving, adaptive coping, and peace of mind. All correlations were statistically significant and aligned with hypothesized relationships.

**Table 2:** Correlation matrix for the variables.

**Variable**	**Hope**	**Academic thriving**	**Adaptive coping**	**Peace of mind**
Hope	1	0.67^***^	0.54^***^	0.53^***^
Academic thriving	0.67^***^	1	0.50^***^	0.59^***^
Adaptive coping	0.54^***^	0.50^***^	1	0.61^***^
Peace of mind	0.53^***^	0.59^***^	0.61^***^	1

^***^p < 0.001.

Hope showed strong positive correlations with academic thriving (*r* = 0.67, *p* < 0.001) and adaptive coping (*r* = 0.54, *p* < 0.001), suggesting higher hope is linked to more positive academic experiences and greater use of effective coping. Academic thriving also correlated positively with adaptive coping (*r* = 0.50, *p* < 0.001), indicating that thriving students tend to employ adaptive strategies. Importantly, adaptive coping strongly correlated with peace of mind (*r* = 0.61, *p* < 0.001). Both hope (*r* = 0.53, *p* < 0.001) and academic thriving (*r* = 0.59, *p* < 0.001) were positively associated with peace of mind, suggesting students with higher levels of these constructs experienced greater emotional balance.

#### 4.1.3 Measurement model

Prior to evaluating structural relationships, a confirmatory factor analysis (CFA) assessed the fit of the measurement model. This model comprised four latent constructs: hope, academic thriving, adaptive coping, and peace of mind, each measured by their respective survey items. The CFA ensured observed variables reliably represented their latent factors.

CFA results indicated a good fit to the data: *CFI* = 0.95, *TLI* = 0.94, *RMSEA* = 0.048 (90% CI:0.042–0.054), and *SRMR* = 0.034. These indices met recommended thresholds (*CFI* and *TLI* ≥ 0.90, *RMSEA* ≤ 0.06, and *SRMR* ≤ 0.08; Hu and Bentler, 1999), suggesting the model adequately captured the covariance structure. Although the chi-square test was significant [$\chi_{\left(120\right)}^{2}$ = 245.32, *p* < 0.001], emphasis was placed on alternative fit indices due to chi-square's sensitivity to large sample sizes (Kline, 2016).

Factor loadings for all observed indicators ranged from 0.65 to 0.88, all statistically significant (*p* < 0.001), confirming items were strong measures of their constructs. Average Variance Extracted (*AVE*) values for all constructs (hope = 0.64, academic thriving = 0.58, adaptive coping = 0.60, peace of mind = 0.62) exceeded the 0.50 threshold, indicating over 50% variance in each indicator was explained by its latent factor (Fornell and Larcker, 1981). Composite Reliability (*CR*) values (hope = 0.88, academic thriving = 0.85, adaptive coping = 0.84, peace of mind = 0.87) were all above 0.70, demonstrating good internal consistency. These results supported the adequacy of the measurement model, allowing progression to structural model analysis.

#### 4.1.4 Structural model

**Figure 1:**
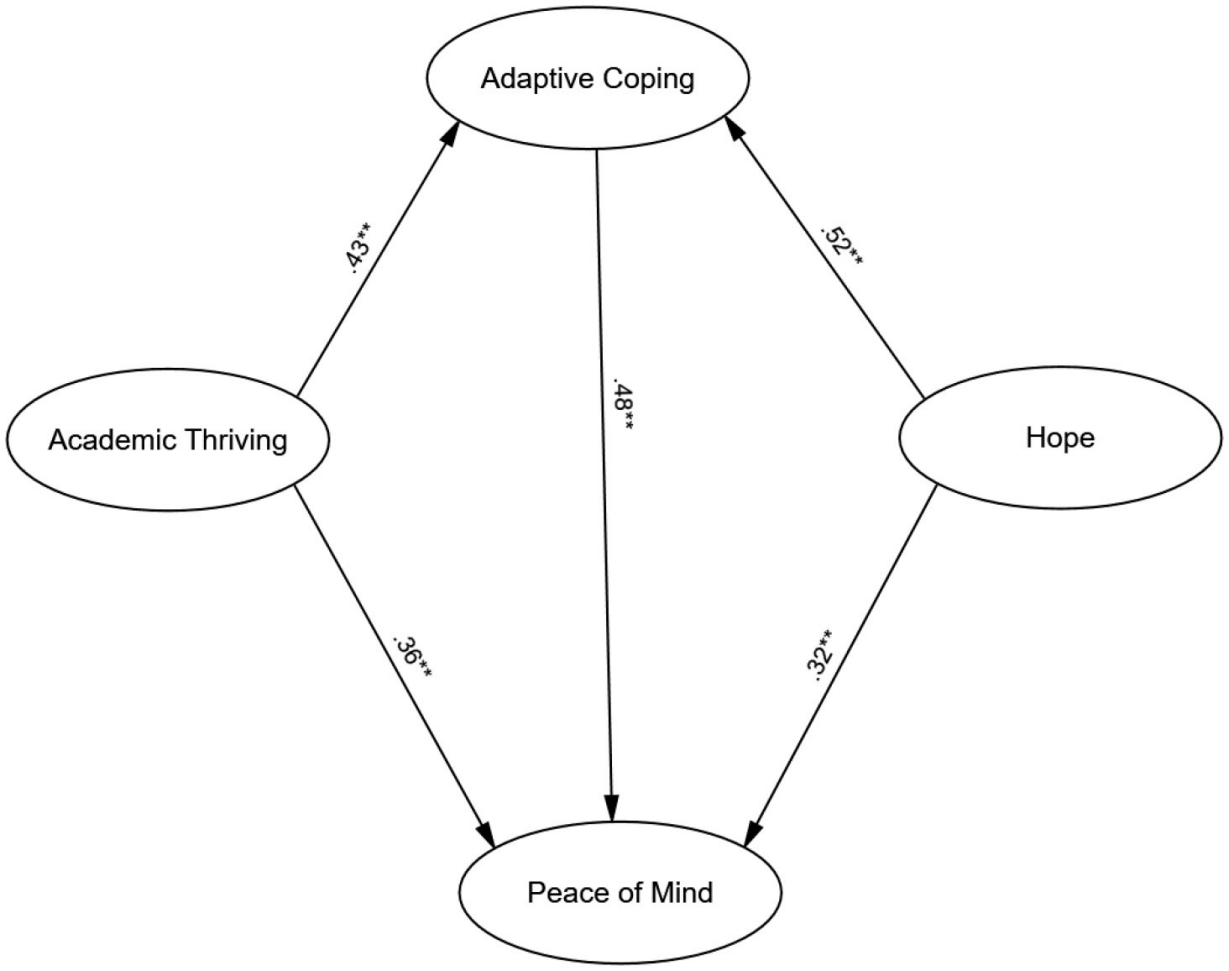
The final model. **p < 0.001.

Following CFA confirmation, Structural Equation Modeling (SEM) in AMOS tested the hypothesized relationships among hope, academic thriving (exogenous variables), adaptive coping (mediator), and peace of mind (outcome). The structural model demonstrated excellent fit: *CFI* = 0.96, *TLI* = 0.95, *RMSEA* = 0.045 (90% *CI*:0.03 −0.052), and *SRMR* = 0.031. These indices indicated good model fit, with the significant chi-square value (χ2(124) = 256.12, *p* < 0.001) interpreted cautiously given its sensitivity to sample size (Kline, 2016). The final model is illustrated in Figure 1.

Standardized path coefficients (Table 3) revealed significant direct effects. Hope significantly predicted adaptive coping (β = 0.52, *p* < 0.001), indicating higher hope levels were linked to increased adaptive coping use. Academic thriving also significantly predicted adaptive coping (β = 0.43, *p* < 0.001), suggesting engaged students employed coping more effectively. For peace of mind, both hope (β = 0.32, *p* < 0.001) and academic thriving (β = 0.36, *p* < 0.001) showed significant direct effects, indicating higher levels of these constructs corresponded with greater emotional balance. Adaptive coping also had a significant direct effect on peace of mind (β = 0.48, *p* < 0.001), underscoring its importance for emotional wellbeing.

**Table 3:** Direct effects in the structural model.

**Path**	**β**	**SE**	***p*-value**
Hope → adaptive coping	0.52	0.04	< 0.001
Academic thriving → adaptive coping	0.43	0.03	< 0.001
Hope → peace of mind	0.32	0.02	< 0.001
Academic thriving → peace of mind	0.36	0.02	< 0.001
Adaptive coping → peace of mind	0.48	0.04	< 0.001

Bootstrapping analysis (5,000 resamples) confirmed adaptive coping significantly mediated the effects of hope (β = 0.25, 95% *CI*: 0.17–0.34, *p* < 0.001) and academic thriving (β =0.21, 95% *CI*:0.14 −0.30, *p* < 0.001) on peace of mind. Substantial total effects for hope (β = 0.57, *p* < 0.001) and academic thriving (β = 0.58, *p* < 0.001) on peace of mind further confirmed their direct and indirect contributions to emotional wellbeing via adaptive coping. The full results of the mediation analysis, including indirect and total effects, are presented in Table 4.

**Table 4:** Indirect and total effects in the structural model.

**Path**	**Indirect effect (β)**	**95% CI**	***p*-value**
Hope → adaptive coping → peace of mind	0.25	0.17–0.34	< 0.001
Academic thriving → adaptive coping → peace of mind	0.21	0.14–0.30	< 0.001
Total effect of hope on peace of mind	0.57	-	< 0.001
Total effect of academic thriving on peace of mind	0.58	-	< 0.001

#### 4.1.5 *Post-hoc* analysis

Given the strong correlation between hope and academic thriving (*r* = 0.67, *p* < 0.001), multicollinearity diagnostics were performed. Variance inflation factors (*VIFs*) for hope and academic thriving were 1.89 and 1.76, respectively, remaining well below the common threshold of 10 (Kline, 2016), indicating no multicollinearity issues.

To ensure construct comparability across demographic groups, measurement invariance analysis was conducted. This process confirms that survey items are interpreted similarly by different subgroups, enabling valid comparisons of structural paths. We assessed invariance across gender, finding good fit for configural invariance (χ2(238) = 458.32, *CFI* = 0.94, *TLI* = 0.92, *RMSEA* = 0.045, *SRMR* = 0.034), confirming equivalent factor structure. Metric invariance was supported by minimal fit deterioration (ΔCFI = −0.002), indicating comparable factor loadings. Scalar invariance also showed acceptable fit (ΔCFI = −0.003), confirming equivalent item intercepts. With configural, metric, and scalar invariance established, constructs were measured equivalently across gender. Subsequent multi-group SEM analysis revealed no significant gender differences in the direct or indirect pathways, suggesting consistent relationships among hope, academic thriving, adaptive coping, and peace of mind for both males and females.

### 4.2 Qualitative results

The qualitative component of this study sought to explore how participants' levels of hope, academic thriving, and their use of adaptive coping strategies influenced their experience of peace of mind, particularly in the context of academic stress. Thirty participants contributed to the qualitative data, with 20 selected for semi-structured interviews and 10 providing reflective journal entries. Thematic analysis of the interviews and journals yielded four overarching themes related to hope, thriving, coping, and emotional wellbeing, providing nuanced insights into the quantitative findings and highlighting how students navigated academic challenges. These themes are detailed below, with integrated participant voices to enrich the discussion.

#### 4.2.1 Hope as a driving force for resilience

Hope emerged as a central factor contributing to participants' resilience in navigating academic challenges, with varying nuances in how students described its role in their coping strategies. Participant 12 (female, third-year student) shared that future-oriented thinking helped her manage overwhelming academic workloads: “*When I felt like everything was piling up, I reminded myself that I've faced tough situations before and made it through. I focused on my long-term goals, which kept me moving forward.”*

This example highlights how hope can offer a pathway to refocusing on larger objectives, enabling students to regain a sense of control over their academic experiences. In contrast, Participant 18 (male, second-year student) emphasized how hope fostered a sense of agency, pushing him to be proactive in problem-solving: “*For me, hope means knowing that there's always a way forward, even when things get tough. It motivates me to keep looking for solutions rather than giving up.”*

This sentiment of persistence was echoed by others, including Participant 25 (female, third-year student), who viewed hope as an emotional anchor: “*During difficult exams, hope is what helps me calm down. It's not just about surviving the moment, but knowing that I have the ability to get through it, no matter what.”*

These varied reflections show that while many participants saw hope as a tool for resilience, the mechanisms through which it functioned differed. For some, hope fueled strategic planning and solution-focused approaches, while for others, it provided emotional support and stability. The diversity of these experiences enriches the overall understanding of hope's role in resilience, moving beyond a one-dimensional portrayal. Additionally, these insights align with literature highlighting hope's capacity to sustain perseverance through both emotional regulation and practical action (Pedrotti, 2018; Snyder et al., 2002).

#### 4.2.2 Academic thriving as engagement and growth

Academic thriving was characterized by participants' deep engagement in learning and personal growth within their academic environment. While grades held importance, many described thriving as something broader and more fulfilling. For example, Participant 7 (male, second-year student) reflected, “*Thriving isn't just about getting the grades. It's about realizing how far I've come—how much more confident, curious, and engaged I am now compared to when I first started university.”* This perspective highlights how thriving encompasses both cognitive and emotional growth, including gains in self-confidence and academic engagement.

Participant 15 (female, third-year student) similarly emphasized the connection between thriving and purpose, stating, “*The more I connect with my studies, the more I feel like I'm thriving. It's about finding purpose in what I'm learning and seeing how it all fits into my bigger goals.”* For many, thriving went beyond academic performance and became linked to emotional fulfillment and a sense of purpose, reinforcing that thriving is rooted in both intellectual engagement and personal growth. This sense of thriving appeared to foster resilience, as students who thrived were more likely to view academic challenges as opportunities for development rather than threats to their success. Other participants echoed this sentiment, illustrating how academic thriving provided not only personal satisfaction but also emotional stability during periods of academic stress.

#### 4.2.3 Adaptive coping as a key mechanism for managing stress

Adaptive coping emerged as a crucial strategy for managing academic stress, with participants sharing a variety of approaches to maintaining balance. Participant 18 (female, second-year student) discussed how breaking tasks into smaller parts and seeking support helped her manage stress: “*When things start piling up, I break everything down into smaller tasks and create a schedule. If I feel overwhelmed, I reach out to friends or professors for help. It keeps me on track and stops me from spiraling.”* This approach reflects how problem-solving and help-seeking behaviors were essential for maintaining control over academic responsibilities.

Additionally, many participants emphasized the importance of balancing academic work with self-care practices. Participant 20 (male, third-year student) shared, “*I make sure to go to the gym a few times a week. Physical activity helps me clear my head and stay focused. It's become part of how I manage my stress.”* The role of physical activity as a coping strategy illustrates how participants actively integrated wellbeing practices into their academic routines to alleviate stress.

Reflective journal entries provided further insights into how coping strategies supported emotional wellbeing. Participant 9 (third-year student) noted, “*Reflecting on how I cope with stress helped me realize that my approach—asking for help when I need it and making time for myself—has kept me grounded. It's not just about getting through tasks, but also about preserving my peace of mind.”* This ability to combine academic work with stress management strategies, such as exercise, social support, and mindfulness, was key in helping participants sustain their emotional wellbeing during stressful periods.

#### 4.2.4 The link between hope, thriving, and peace of mind

The participants described a dynamic relationship between hope, academic thriving, and peace of mind, with each factor contributing to their overall emotional wellbeing. Hope was often seen as the foundation of resilience, helping students persevere through academic challenges, while thriving in their academic endeavors provided a sense of purpose and fulfillment. These factors worked together to help participants maintain emotional balance.

For instance, Participant 25 (male, third-year student) reflected on the calming influence of hope: “*When I feel hopeful about my future and see progress in my studies, I'm more relaxed. It's like, no matter what happens, I know things will turn out okay, and that gives me peace of mind.”* This highlights how the combination of hope and academic progress contributed to a sense of mental clarity and emotional stability.

Similarly, several participants noted that thriving academically helped them manage stress more effectively. Participant 14 (female, second-year student) explained, “*I've noticed that when I'm doing well academically, I feel much calmer. It's easier to manage stress because I don't feel like everything is falling apart.”* This demonstrates how a sense of academic achievement can foster emotional calmness and clarity, preventing stress from becoming overwhelming.

Participants who reported higher levels of peace of mind frequently mentioned the use of adaptive coping strategies, suggesting that effective coping played a key role in maintaining emotional stability. Many emphasized that thriving academically and maintaining hope enabled them to approach stress with a clearer, more balanced perspective. This interplay between hope, thriving, and adaptive coping created a feedback loop, where positive academic experiences reinforced emotional wellbeing, which in turn, allowed participants to continue thriving in their academic environments.

## 5 Discussion

This mixed-methods study explored the relationships between hope, academic thriving, adaptive coping, and peace of mind among university students. The findings offer insights into how these specific psychological resources, such as hope and thriving, contribute to students' emotional wellbeing, with adaptive coping playing a mediating role. The discussion interprets these results within the context of existing literature and theoretical frameworks, emphasizing the unique contributions and distinct nature of each construct.

The first hypothesis (H1), proposing a direct relationship between hope and peace of mind, was strongly supported by the data. Consistent with Snyder's Hope Theory 2002, hope, through its components of agency and pathways, emerged as a key resource for managing stress and promoting emotional balance (Gallagher et al., 2017; Pedrotti, 2018). This finding highlights hope not merely as a positive affect or a generalized optimistic outlook, but as a deliberate cognitive-motivational disposition where individuals proactively generate solutions and maintain resolve toward goals. Hopeful students tend to view academic challenges as manageable, fostering resilience and emotional calm (Rand et al., 2020; Yoon et al., 2015). These findings align with studies emphasizing hope's role in stress management and emotional regulation (Bryce et al., 2020; Marques et al., 2017). The qualitative data further supported these results, with participants describing hope as a motivational force that enabled them to persevere through academic difficulties, which is consistent with the literature on perseverance and goal achievement (Fraser et al., 2022a; Levi et al., 2014).

The second hypothesis (H2), which posited that academic thriving is directly related to peace of mind, was also supported. Thriving students—who are engaged in their learning, foster social connections, and maintain a positive outlook—reported higher emotional wellbeing. This finding aligns with Schreiner's (2010) concept of thriving, which integrates cognitive, emotional, and social dimensions to support student wellbeing (Cuevas et al., 2017; Rodríguez-Fernández et al., 2018). This “thriving” is distinct from general wellbeing by being specifically rooted in the academic context, encompassing active engagement and growth within the university environment. It moves beyond merely academic performance by integrating a student's emotional and social flourishing within their educational journey (Schreiner, 2010; Cuevas et al., 2017). Thriving students are not only academically successful but also emotionally resilient, allowing them to better manage stress and recover from setbacks (Green and Rizwan, 2024; Morgan Consoli et al., 2015). The qualitative data reinforced this, with participants describing how their thriving experiences contributed to personal growth and emotional fulfillment, supporting the view that thriving extends beyond academic performance (Kristjánsson, 2016; Sayler, 2009).

The third and fourth hypotheses (H3 and H4), examining adaptive coping's mediating role between hope, academic thriving, and peace of mind, were supported. As discussed, coping strategies are considered adaptive within a specific context based on their beneficial outcomes. Adaptive coping strategies (e.g., problem-solving, help-seeking, emotional regulation) were crucial mechanisms through which hope and thriving contributed to peace of mind (Skinner et al., 2016; Vizoso et al., 2018). These findings align with literature emphasizing adaptive coping's importance in managing academic stress and promoting psychological wellbeing (Enns et al., 2018; Gloria and Steinhardt, 2016). Adaptive coping enables emotional balance by proactive responses to stressors, preventing exhaustion (Halland et al., 2015; Wu et al., 2020). Hopeful students, grounded in positive outcomes and goal-directed thinking, tend to employ adaptive strategies like problem-solving and help-seeking (Snyder et al., 2002; Fraser et al., 2022b), demonstrating higher emotional regulation capacity, which fosters stability and peace of mind (Marques et al., 2017; Yoon et al., 2015). Similarly, thriving students, deeply engaged with their academic and social environments, are more likely to employ adaptive coping (Schreiner, 2010; Rodríguez-Fernández et al., 2018). Their engagement, social connectedness, and positive outlook equip them with resources to manage academic pressures (Green and Rizwan, 2024; Kotzé and Kleynhans, 2013). Adaptive coping thus bridges academic success and emotional wellbeing, allowing thriving students to sustain both performance and peace of mind (Cuevas et al., 2017; Vizoso et al., 2018). Qualitative findings further revealed participants' reliance on adaptive coping strategies (e.g., breaking down tasks, seeking support, self-care) for emotional balance, aligning with research on mindfulness and emotional regulation reducing stress (Halland et al., 2015; Gloria and Steinhardt, 2016). These strategies preserve peace of mind and strengthen resilience (Fraser et al., 2022a; Enns et al., 2018).

A key contribution of this study is the identification and elaboration of the synergistic relationship between hope and academic thriving. Our results demonstrated a strong positive correlation between these two constructs (*r* = 0.67, *p* < 0.001), indicating their close intertwining. While both hope (β = 0.32) and academic thriving (β = 0.36) showed significant independent direct effects on peace of mind, their substantial correlation and shared contribution to adaptive coping suggest a mutually reinforcing dynamic. Hope, with its focus on goal-directed agency and pathways, can drive the engaged learning and determination characteristic of academic thriving. Conversely, experiencing success and positive engagement inherent in thriving can reinforce a student's belief in future goals, thereby strengthening their hope. This interconnectedness means students leverage a composite psychological resource where each component amplifies the other, leading to a greater likelihood of adopting effective coping strategies (Carmona-Halty et al., 2021). This synergy is distinct from coping, as hope and thriving provide the motivational base, while coping involves the behavioral application; it also differs from general wellbeing by focusing on academic-specific growth. This, in turn, enables them to manage academic stress in ways that preserve their peace of mind (Schreiner, 2010; Snyder et al., 2002; Rodríguez-Fernández et al., 2018). The ability to employ adaptive coping strategies—such as reframing challenges, problem-solving, and emotional regulation—creates a feedback loop where emotional wellbeing enhances hope and thriving, further reinforcing students' resilience and emotional stability (Bryce et al., 2020; Fraser et al., 2022b).

This cyclical nature of psychological resilience underscores the importance of fostering both cognitive and emotional resources in academic settings. When students are equipped with adaptive coping skills and supported in developing hope and thriving behaviors, they are better able to sustain emotional balance and perform well academically, even in high-pressure environments (Datu et al., 2018; Wu et al., 2020). Thus, the study's findings highlight the dynamic interaction between these constructs, offering practical implications for interventions aimed at enhancing student wellbeing through the cultivation of hope, thriving, and adaptive coping strategies (Snyder et al., 2002; Rodríguez-Fernández et al., 2018; Schreiner, 2010).

Overall, this study underscores the critical role of hope, academic thriving, and adaptive coping in fostering peace of mind among university students. These psychological resources not only contribute directly to emotional wellbeing but also work through adaptive coping to buffer against academic stress. The findings highlight the need to support students in developing hope and thriving behaviors and equipping them with adaptive coping strategies. Future research should continue to explore these complex interactions, particularly in diverse academic and cultural contexts (Lee et al., 2013; Liao et al., 2023).

## 6 Conclusion and implications

This study explored the relationships between hope, academic thriving, adaptive coping, and peace of mind among university students facing academic stress. Our findings supported the conceptual model, demonstrating that hope and academic thriving significantly predict peace of mind, both directly and through the mediation of adaptive coping strategies. This underscores the importance of fostering these psychological resources and adaptive coping mechanisms to effectively manage academic challenges. Our mixed-methods approach further enriched this understanding by integrating quantitative findings with qualitative insights into students' personal experiences, illustrating how they navigate academic pressures with hope, resilience, and adaptive strategies.

Theoretically, this study contributes to the fields of positive psychology, educational psychology, and stress management by integrating hope theory (Snyder, 2002), the academic thriving model (Schreiner, 2010), and adaptive coping frameworks (Skinner et al., 2013). It builds on existing theories by demonstrating how these constructs interact to promote peace of mind, a relatively understudied aspect of emotional wellbeing in academic settings. The study extends hope theory by confirming the mediating role of adaptive coping strategies, showing that hope's influence on peace of mind is both direct and indirect through constructive behaviors. Similarly, it expands the academic thriving model by emphasizing the role of coping in sustaining both academic success and emotional balance.

Moreover, this research advances the literature by exploring peace of mind as a distinct psychological construct in academic contexts, where the focus has traditionally been on higher-arousal emotional states, such as happiness or satisfaction. The finding that peace of mind is significantly associated with both hope and thriving adds a new dimension to understanding how students navigate academic challenges while maintaining psychological equilibrium. This study also opens pathways for future research on emotional wellbeing by proposing adaptive coping as a critical mechanism that can facilitate the attainment of peace of mind in stressful environments.

This research has significant practical implications for educators, counselors, and university administrators focused on enhancing student wellbeing and academic resilience. One of the central findings suggests that institutions should focus on promoting hope by implementing programs and workshops aimed at cultivating goal-setting skills, boosting self-efficacy, and helping students devise strategies for overcoming academic obstacles. For instance, university counseling services could develop “Hope-Focused Mentoring Programs” that pair senior students or faculty with mentees to guide them in setting realistic academic and personal goals, anticipating potential challenges, and collaboratively brainstorming multiple pathways to success. Academic skills centers could offer “Resilience-Building Workshops” focused on teaching students how to break down large tasks into manageable steps and develop contingency plans, thereby reinforcing their sense of agency and pathway thinking (Snyder, 2002; Pedrotti, 2018). By fostering a sense of hope, students can develop a forward-thinking mindset that empowers them to face academic challenges with greater confidence and resilience.

In addition to promoting hope, encouraging academic thriving is vital. This can be achieved by creating supportive, engaging learning environments that not only prioritize intellectual growth but also nurture emotional and social wellbeing. Universities should actively promote social connectedness by facilitating meaningful relationships between students, their peers, and faculty members. Concrete strategies include establishing structured “Learning Communities” for first and second-year students within specific disciplines, offering “Faculty-Student Research Opportunities” that foster deep intellectual engagement and mentorship, and organizing “Interdisciplinary Project-Based Learning Initiatives” that encourage collaborative problem-solving. To boost social connectedness, counseling services and student affairs offices could co-create “Peer-Mentoring Circles” or facilitate “Student Interest Groups” linked to academic success, providing platforms for emotional support and shared academic identity (Schreiner, 2010; Rodríguez-Fernández et al., 2018). By encouraging active engagement in learning and fostering a sense of belonging, institutions can help students discover personal relevance in their studies, which ultimately enhances both academic success and emotional fulfillment.

Teaching adaptive coping strategies is another essential recommendation. These strategies should be explicitly integrated into academic settings, with a particular focus on problem-solving, emotional regulation, and help-seeking behaviors. University counseling services could offer regular “Coping Skills Seminars” that cover practical techniques for managing academic pressure, such as effective time management, cognitive restructuring (reframing negative thoughts), and emotion regulation exercises. Specialized programs, like “Mindfulness-Based Stress Reduction (MBSR) adaptations for students” or “Emotional Intelligence (EI) training for academic success,” could be introduced, recognizing that enhanced emotional competencies are crucial for navigating complex academic and professional demands (Tripon, 2023). Furthermore, integrating brief modules on adaptive coping into large lecture courses or orientation programs could ensure broad reach, while peer support groups and academic mentoring programs can provide valuable social resources for practicing help-seeking behaviors (Skinner et al., 2013; Wu et al., 2020). By reinforcing adaptive coping behaviors, universities can equip students with the skills needed to manage stress effectively, thus fostering both academic persistence and mental health.

Lastly, fostering peace of mind should be a key priority for universities. Recognizing peace of mind as a crucial psychological resource, institutions should implement initiatives that promote emotional tranquility. Providing access to mental health services, including counseling and stress management programs, is essential in this regard. Specific initiatives could include creating “Calm Corners” or designated quiet spaces on campus, offering “Guided Relaxation Podcasts” or virtual mindfulness sessions accessible to all students, and organizing “Nature-Based Wellness Walks” or “Campus Yoga/Meditation Sessions.” University counseling centers could also expand brief, solution-focused counseling models specifically targeting stress reduction and emotional balance, aiming to cultivate students' inner calm and mental clarity amidst academic pressures (Lee et al., 2013; Xu et al., 2015). By addressing these factors, universities can create an academic environment that not only supports students' intellectual achievements but also sustains their emotional wellbeing.

## 7 Limitations and suggestions for future research

This study has several limitations that should be acknowledged. First, the cross-sectional design limits the ability to establish causal relationships between the variables. While structural equation modeling provided support for the hypothesized pathways, longitudinal research is needed to confirm the directionality of these relationships and understand how hope, thriving, coping, and peace of mind evolve over time. Future longitudinal studies could employ repeated measures throughout an academic year, for example, collecting data at the beginning, middle, and end of a semester to observe changes in hope, thriving, and peace of mind as academic demands fluctuate, and to track the development of adaptive coping strategies over time. This would allow for stronger inferences about the causal pathways.

Second, the study was conducted within a specific cultural context—mainland China—which may affect the generalizability of the findings to other cultural or educational settings. Given that peace of mind is deeply rooted in Eastern philosophies (Lee et al., 2013), future research should explore whether similar relationships are observed in Western or other cultural contexts, where different emotional and psychological frameworks may shape students' coping strategies and wellbeing. Specifically, comparative studies could examine how cultural dimensions (e.g., individualism-collectivism, power distance) moderate the roles of social support in coping, or how academic thriving manifests in educational systems with different pedagogical approaches. Cross-cultural comparisons could provide valuable insights into how the constructs of hope, thriving, coping, and peace of mind function across various educational systems and cultural expectations.

Third, the study included participants from a wide range of academic disciplines, such as engineering, social sciences, and humanities. While this approach aimed to enhance the generalizability of our findings to the broader university student population, we acknowledge that different academic fields may present unique demands and require specific psychological resources, including varying cognitive and emotional skill sets. Our current analysis did not disaggregate findings by academic discipline. Future research could specifically investigate potential variations in the relationships between hope, academic thriving, adaptive coping, and peace of mind across distinct academic programs (e.g., comparing STEM students with humanities students). This could involve multi-group SEM analyses or qualitative deep dives into discipline-specific stressors and coping resources, offering a more nuanced understanding of student wellbeing within specialized higher education contexts.

Fourth, although well-established self-report scales were used to measure the constructs of interest, such data may be subject to biases like social desirability or recall bias. Future studies could benefit from incorporating more objective measures of coping strategies, academic performance, and psychological wellbeing. For example, incorporating physiological indicators of stress (e.g., salivary cortisol levels before and after exams), observer ratings of engagement, or real-time ecological momentary assessment (EMA) to capture coping behaviors in daily academic life could provide a more detailed and less biased understanding of how students cope with academic stress in naturalistic settings.

Finally, this study focused on adaptive coping strategies as mediators. Future research should consider additional factors that may influence the relationship between hope, thriving, and peace of mind. Personality traits such as resilience or grit, as well as external factors like family support or financial stress, may also play significant roles in shaping students' emotional wellbeing. Investigating these factors could lead to a more nuanced understanding of how students navigate academic challenges and maintain their mental health. Furthermore, future research could specifically leverage insights from affective neuroscience and advanced neuroimaging techniques to explore the precise neural mechanisms underlying the experience and cultivation of peace of mind in academic contexts, potentially examining brain activity during mindfulness interventions designed to enhance calmness. Future studies should also explore the effectiveness of specific interventions derived from these findings, such as hope-enhancement programs, academic thriving workshops, or tailored adaptive coping skill training, and assess their long-term impact on students' peace of mind and overall wellbeing.

## Data Availability

The data analyzed in this study is subject to the following licenses/restrictions: the datasets used and/or analyzed during the current study are available from the corresponding author upon reasonable request. Requests to access these datasets should be directed to Jing Shan, qq7448780@sina.com.
